# Supplementary material for: Prediction of Microstructural Representativity From A Single Image
Source: Adv Sci (Weinh). 2025 Jul 23;12(34):e14149. doi: 10.1002/advs.202414149 (PMC12442635; doi:10.1002/advs.202414149)
Supplement: Supplementary file 1 — Supporting Information [file ADVS-12-e14149-s001.pdf]

ADVANCED SCIENCE
Open Access

Supporting Information

for *Adv. Sci.*, DOI 10.1002/advs.202414149

Prediction of Microstructural Representativity From A Single Image

Amir Dahari, Ronan Docherty, Steve Kench and Samuel J. Cooper*

ImageRep: Predicting microstructural representativity from a single image - Supplementary Information

Amir Dahari [1], Ronan Docherty [1,2], Steve Kench [1], and Samuel J. Cooper [1]

[1]*Dyson School of Design Engineering, Imperial College London, London SW7 2DB*

[2]*Department of Materials, Imperial College London, London SW7 2AZ*

Contents

1 Calculating periodic TPC using FFT **2**

2 Generating random spheres on a plane with a given phase fraction expected value **3**

3 Finding r_0 in practice **4**

4 Variation prediction using periodic TPC **4**

4.1 Other changes for enhanced stability results . . . 5

5 Validation information breakdown **5**

5.1 PoreSpy generators used . . . 5

5.2 SOFC void defect inpainting . . . 5

5.3 Image sizes used for validation . . . 7

5.4 Classical one image subdivision method . . . 8

5.5 Validation including only std prediction step . . . 8

6 Discussion SI **9**

6.1 Impact of phase fraction uncertainty on battery performance . . . 9

6.2 Assessing image unrepresentativeness . . . 9

1 Calculating periodic TPC using FFT

The proof of the connection between the periodic Two-Point Correlation function (TPC) and the Discrete Fourier Transform (DFT) can be found in [1] (chapter 12.3.1). We present it here using our notation, and expanding on some calculations for clarity. The fact that the TPC can be calculated using DFT allows for a faster calculation using the Fast Fourier Transform (FFT) algorithm. If the image size is $|X|$, then the number of calculations for calculating the TPC drops from an order of $|X|^2$ to an order of $|X| \cdot log(|X|)$. This is a dramatic change, for example if X is a 1000^3 volume, the number of computations drop from $1 \cdot 10^{18}$ to $2 \cdot 10^{10}$.

From this point forward, we drop the image ω notation for simplicity, as all calculations are made on a specific image. As in [1], we provide the proof in 1D, and the extension to higher dimensions is straight-forward. In Section 3.1, the non-periodic TPC was defined. Here, we work with the periodic TPC, which we define by $T^p_{r,X}$ instead of $T_{r,X}$, where the multiplications are "wrapped around" and the indices are indexed mod image length. For example in the simple case of 1D,

$$T^p_{r,X} = \frac{1}{|X|} \sum_{x=0}^{|X|-1} B_x \cdot B_{x+r} \tag{1}$$

and if $x + r \geq |X|$, then $x + r = x + r - |X|$ (mod $|X|$).

The DFT of order $0 \leq k \leq |X| - 1$ for the sequence of numbers $\boldsymbol{B} = \{B_0, B_1, \dots B_{|X|-1}\}$ is

$$\mathcal{F}_k(\boldsymbol{B}) = \sum_{x=0}^{|X|-1} B_x \cdot e^{\frac{-2\pi xk}{|X|}}$$

On the other hand, if we define the sequence of all TPC's as $\boldsymbol{T^p_X} = \{T^p_{0,X}, T^p_{1,X}, \dots, T^p_{|X|-1,X}\}$, then

$$\mathcal{F}_k(\boldsymbol{T^p_X}) = \sum_{y=0}^{|X|-1} \frac{1}{|X|} \sum_{x=0}^{|X|-1} B_x \cdot B_{x+y} \cdot e^{\frac{-2\pi yk}{|X|}} =$$

$$= \frac{1}{|X|} \sum_{y=0}^{|X|-1} B_x \sum_{x=0}^{|X|-1} \cdot B_{x+y} \cdot e^{\frac{-2\pi yk}{|X|}}$$

If we set $z = y + x$, then

$$\mathcal{F}_k(\boldsymbol{T^p_X}) = \frac{1}{|X|} \sum_{y=0}^{|X|-1} B_x \sum_{z=y}^{y+|X|-1} \cdot B_t \cdot e^{\frac{-2\pi(z-x)k}{|X|}} =$$

and since the indices are periodic, the summation of z from $z = y$ to $z = y + |X| - 1$ is the same as the summation of z from 0 to $|X| - 1$. In addition to taking one exponent outside of the inner sum result in

$$\mathcal{F}_k(\boldsymbol{T^p_X}) = \frac{1}{|X|} \sum_{y=0}^{|X|-1} B_x \cdot e^{\frac{2\pi xk}{|X|}} \sum_{z=0}^{|X|-1} \cdot B_z \cdot e^{\frac{-2\pi zk}{|X|}} =$$

so,

$$\mathcal{F}_k(\boldsymbol{T^p_X}) = \frac{1}{|X|} \mathcal{F}_k(\boldsymbol{B}^*) \mathcal{F}_k(\boldsymbol{B}) \tag{2}$$

where $\boldsymbol{B}^*$ is the complex conjugate of $\boldsymbol{B}$.

Equation (2) holds for every $0 \geq k \geq |X| - 1$, so also

$$\frac{1}{|X|}\sum_{k=0}^{|X|-1}\mathcal{F}_k(\boldsymbol{T^p_X})\cdot e^{\frac{2\pi rk}{|X|}} = \frac{1}{|X|^2}\sum_{k=0}^{|X|-1}\mathcal{F}_k(\boldsymbol{B}^*)\cdot\mathcal{F}_k(\boldsymbol{B})\cdot e^{\frac{2\pi rk}{|X|}} \tag{3}$$

The left hand side is exactly the periodic TPC with distance r : $T^p_{r,X}$ which follows from the inverse DFT definition, and the right hand side is

$$T^p_{r,X} = \frac{1}{|X|}\mathcal{F}_r^{-1}\left(\boldsymbol{\mathcal{F}}(\boldsymbol{B}^*)\cdot\boldsymbol{\mathcal{F}}(\boldsymbol{B})\right) \tag{4}$$

for the sequence

$$\boldsymbol{\mathcal{F}}(\boldsymbol{B}^*)\cdot\boldsymbol{\mathcal{F}}(\boldsymbol{B}) = \left(\mathcal{F}_0(\boldsymbol{B}^*)\cdot\mathcal{F}_0(\boldsymbol{B}),\ldots,\mathcal{F}_{|X|-1}(\boldsymbol{B}^*)\cdot\mathcal{F}_{|X|-1}(\boldsymbol{B})\right)$$

Therefore, calculating the TPC requires calculating the DFT (or the inverse DFT) of the image only 3 times using the FFT algorithm using Equation 4. The extension to 2 and 3 dimensions is straight-forward - simply more summations in each equation.

2 Generating random spheres on a plane with a given phase fraction expected value

In Figure 2 of the main paper, a method for creating circles on a plane is presented. Importantly, given a desired phase fraction, the method generates circles such that the expected value of a random image matches the given phase fraction.

Given a desired phase fraction ϕ, circle radius r and image size $l \times l$, the algorithm for creating a random image is:

1. Create a $(l+2r)\times(l+2r)$ image of independently uniform random numbers between 0 and 1.
2. Every pixel can be the center of a circle (the circles can overlap), a pixel is independently chosen to be the center of a circle if the probability value in that pixel is under

   $$1-(1-\phi)^{\frac{1}{\pi r^2}}$$

3. Create circles around successful pixels (1 values) and all other values turn to 0.
4. Crop the center $l \times l$ image.

To prove that the expected value of the phase fraction from this random generation is ϕ, we need to show that the expected value of each pixel is ϕ. A pixel takes the value 1 if it belongs to any circle, whether as the center or part of another circle. The probability that a pixel does not belong to any circle equals the probability that none of the surrounding πr^2 pixels are chosen as circle centers. The probability that a single pixel is not the center of a circle is

$$(1-\phi)^{\frac{1}{\pi r^2}}$$

Since the selection of centers is independent, the probability that a pixel has a value of 0 (i.e., does not belong to any circle) is:

$$\left((1-\phi)^{\frac{1}{\pi r^2}}\right)^{\pi r^2} = 1-\phi$$

And the complement probability of a pixel to be 1 is ϕ. The last crop step ensures that all pixels have the same probability to be in a circle.

3 Finding r_0 in practice

When analyzing a single image of a macro-homogeneous material, the threshold distance r_0, as discussed in Section 3 of the main paper, cannot be determined in advance. This threshold represents the distance beyond which data points are (almost) uncorrelated.

In practice, we select r_0 from a set of distances in increments of 100 pixels, i.e., $r_0 \in 100, 200, ...$ We define r_0 as the smallest distance such that, for a ring in the TPC function (e.g., between 200 and 300 TPC distances), the TPC values deviate from ϕ^2 by more than $0.05 \cdot (\phi - \phi^2)$ in fewer than 3% of the cases. This ensures that the signal to noise ratio is high and that we integrate valuable information from the TPC function. The increments of 100 are chosen for consistency measurements between different images of the same material.

Understanding the impact of r_0 on the final representativity prediction may be useful for improving the method in a further studies. Its selection may be improved by applying a median filter to the TPC curve, which could help reduce noise and yield a more robust estimate. Exploring such techniques could make the identification of r_0 more stable, especially when analyzing single images.

4 Variation prediction using periodic TPC

To use the fast computation of the FFT for the TPC results, we changed the prediction of the variation to use periodic TPC. Specifically, using the same notation as in Section 3 in the main paper,

Theorem 4.1. *For large enough* r_0 *and appropriate constant* $C^p_{r_0}$*, if we set* $\Psi^p : \Omega \to \mathbb{R}$ *to be*

$$\Psi^p(\omega) = \frac{1}{C^p_{r_0}} \cdot \sum_{r \leq r_0} \left(T^p_{r,X}(\omega) - \Phi^2(\omega)\right)$$

then $E[\Psi^p] = Var[\Phi]$.

Proof. Every periodic TPC can be divided into two parts, one part which is the non-periodic TPC and one part who "wraps" around and has long-range correlations (if the TPC vector is shorter than half the image size). I. e. if the complement periodic vector to r is r_p, then

$$T^p_{r,X} = \frac{|X_r|}{|X|} \cdot T_{r,X} + \frac{|X| - |X_r|}{|X|} \cdot T_{r^p,X}$$

So

$$\sum_{r \leq r_0} \left(T^p_{r,X} - \Phi^2\right) = \tag{5}$$

$$= \sum_{r \leq r_0} \left(\frac{|X_r|}{|X|} \cdot T_{r,X} + \frac{|X| - |X_r|}{|X|} \cdot T_{r^p,X} - \Phi^2\right) = \tag{6}$$

$$= \sum_{r \leq r_0} \frac{|X_r|}{|X|} \cdot \left(T_{r,X} - \Phi^2\right) + \sum_{r \leq r_0} \frac{|X| - |X_r|}{|X|} \cdot \left(T_{r^p,X} - \Phi^2\right) \tag{7}$$

In expectation, the left additive part of Equation (7) is exactly $C_{r_0} \cdot Var[\Phi]$ by the theorem in Section 3 in the main paper. So taking the expectation of both sides (Equations (5) and (7)) yields

$$\sum_{r \leq r_0} \left(E[T^p_{r,X}] - E[\Phi^2]\right) = C_{r_0} \cdot Var[\Phi] + \sum_{r \leq r_0} \frac{|X| - |X_r|}{|X|} \cdot \left(E[T_{r^p,X}] - E[\Phi^2]\right) \tag{8}$$

We assume that the image is large enough such that r_0 is much smaller then half of the image size, which means that for all $r \leq r_0,\ r^p >> r_0$ and so $E[T_{r^p,X}] = E[\Phi]^2$ by the macro-homogeneous assumption presented in Section 3 in the main paper. Thus, since $Var[\Phi] = E[\Phi]^2 - E[\Phi]^2$, Equation (8) becomes

$$\sum_{r\leq r_0} \left(E[T^p_{r,X}] - E[\Phi^2]\right) = \left(C_{r_0} + \sum_{r\leq r_0} \frac{|X| - |X_r|}{|X|}\right) \cdot Var[\Phi] \tag{9}$$

and by setting

$$C^p_{r_0} = C_{r_0} + \sum_{r\leq r_0} \frac{|X| - |X_r|}{|X|}$$

we reach the required result.

□

4.1 Other changes for enhanced stability results

We've made two changes to the prediction presented by Ψ^p presented in Theorem 4.1, which help stabilise the output predictions:

1. instead of using Φ^2 in the equation of Theorem 4.1, we normalise this value with the TPC values that are close to r_0. Specifically, If

   $$R_0 = \{r\ :\ |r_0 - 10| \leq |r| \leq |r_0|\}$$

   then this value is defined by

   $$\tilde{T}_{r_0,X} = \frac{1}{|R_0|} \sum_{r\in R_0} T_{r,X}$$

   and the value used instead of Φ^2 is a mean of these two values,

   $$\frac{\Phi^2 + \tilde{T}_{r_0,X}}{2}$$

   This small change might help with stability by decreasing the sensitivity of the predictions to the image phase fraction, but a further study into this stabilisation is needed.
2. Another implementation for stability is by comparing the model's prediction to that of the one-image subdivision method. The one-image subdivision method is computationally lighter than our model and does not significantly impact computation speed. If the model's prediction is more than three times higher or less than one-third of the subdivision method's prediction, the TPC values are adjusted to be more positive or more negative, respectively. In practice, this adjustment is only applied to very small images.

5 Validation information breakdown

5.1 PoreSpy generators used

Table 1 shows the 50 different PoreSpy [4] generators used, that are all the combinations of parameters in the table. For example, a Fractal Noise generator was used with the parameters "frequency"=0.025, "octaves"=12 and "mode"="value". There are $5 \cdot 3 \cdot 2 = 30$ Fractal Noise generators and $5 \cdot 4 = 20$ Blobs generators.

5.2 SOFC void defect inpainting

The SOFC anode dataset [2] is a large 3D dataset of a 3-phase material. The dataset was used for 2D validation since one of the directional axis is small (the anode is not thick enough). For independence between the chosen

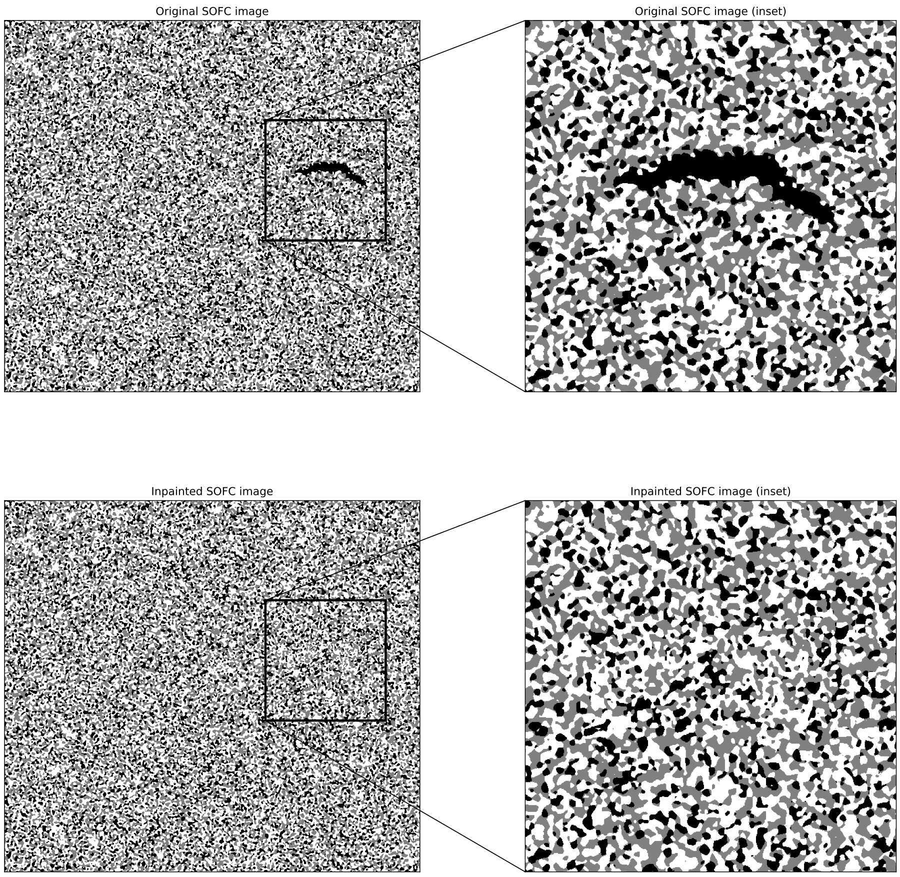

Figure 1: The origianl 2D slice (number 46) of the SOFC anode [2], the inpainted image used for validation and the void defect inpainted area. We used [3] for the inpainting.

Table 1: Breakdown of generators used from PoreSpy [4].

Generator kind		Param 1	Param 2	Param 3
Fractal Noise	Param name	”frequency”	”octaves”	”mode”
	Option set	{0.015, 0.025, 0.035, 0.045, 0.055}	{2, 7, 12}	{”simplex”, ”value”}
Blobs	Param name	”blobiness”	”porosity”	
	Option set	{l/50, l/100, l/150, l/200, l/250} for l = average length of image.	{0.1, 0.2, 0.3, 0.4}	

slices, we picked 4 slices with 23 voxels distance between them (slice 46, 69, 92 and 115). This seems like

a reasonable choice that would produce extra meaningful validation information given that the characteristic length scale of the different phases is between 7 and 9. The dataset has a local void defect that would harm the validation results. The defect is local and every sample from that region will have an extreme phase fraction that will be very different from the bulk phase fraction. We've decided to inpaint this region to not lose a large section from the image due to cropping the image. Figure 1 shows how the inpainting was carried out to slice 46.

Table 2: Breakdown of all validation results

Method type	Material's true phase fraction is inside the predicted bounds	Confidence goal	Absolute error
PoreSpy materials			
Subdivision method (2D)	9238/10000 = 92.38%	95%	2.62%
ImageRep only std (2D)	9404/10000 = 94.00%	95%	0.96%
**ImageRep (2D)**	9442/10000 = 94.42%	95%	**0.58%**
Subdivision method (3D)	914/1000 = 91.40%	95%	3.60%
ImageRep only std (3D)	930/1000 = 93.00%	95%	2.00%
**ImageRep (3D)**	941/1000 = 94.10%	95%	**0.90%**
Solid Oxide Fuel Cell anode			
Subdivision method (2D)	967/1008 = 95.93%	95%	0.93%
ImageRep only std (2D)	943/1008 = 93.55%	95%	1.45%
**ImageRep (2D)**	956/1008 = 94.84%	95%	**0.16%**
Targray battery separator			
Subdivision method (3D)	142/200 = 71%	95%	24.00%
**ImageRep only std (3D)**	189/200 = 94.5%	95%	**0.50%**
**ImageRep (3D)**	191/200 = 95.5%	95%	**0.50%**
PP165 battery separator			
Subdivision method (3D)	200/200 = 100%	95%	5.00%
**ImageRep only std (3D)**	192/200 = 96%	95%	**1.00%**
**ImageRep (3D)**	192/200 = 96%	95%	**1.00%**
All materials			
Subdivision method (2D)	10205/11008 = 92.71%	95%	2.29%
ImageRep only std (2D)	10347/11008 = 94.00%	95%	1.00%
**ImageRep (2D)**	10398/11008 = 94.46%	95%	**0.54%**
Subdivision method (3D)	1256/1400 = 89.71%	95%	5.29%
ImageRep only std (3D)	1311/1400 = 93.64%	95%	1.36%
**ImageRep (3D)**	1324/1400 = 94.57%	95%	**0.43%**

5.3 Image sizes used for validation

Different image sizes were used for validation. In 2D, for each PoreSpy generator, for each image size $\{600^2, 800^2, 1000^2, 1200^2, 1400^2\}$, 40 images were randomly sampled from the generated materials, totalling in 200 samples per material. For 3D, because of the longer computation time, for each image size $\{280^3, 310^3, 340^3, 370^3, 400^3\}$, 4 images were randomly sampled from the generated materials, totalling in 20 samples per material.

For the SOFC material, for each of the 3 phases, smaller images were sampled $\{400^2, 500^2, 600^2\}$ and also for the 3D separators $\{230, 260, 290, 320\}$ since the experimental microstructures are smaller than the PoreSpy simulated ones.

5.4 Classical one image subdivision method

For applying the subdivision method for a single image, we apply the same procedure as in [5, 6]. The image is progressively divided into smaller subsamples, and for each set of subsamples of the same size, we calculate the standard deviation of their phase fractions.

After computing standard deviations for different sizes, the characteristic length scale (or integral range) is found by fitting the best integral range in Equation (2) presented in Section 2 of the main text.

Specifically, the image is divided into ratios $2, 4, 8, \ldots$ while ratio 2 divides a 2D image into 4 and a 3D image into 8, ratio 4 divides a 2D image into 16 and a 3D image into 64, and so on. Very small subsamples such as 1, 2, 3 or 4 pixels/voxels are not used as their phase fractions are very unstable with very high standard deviations.

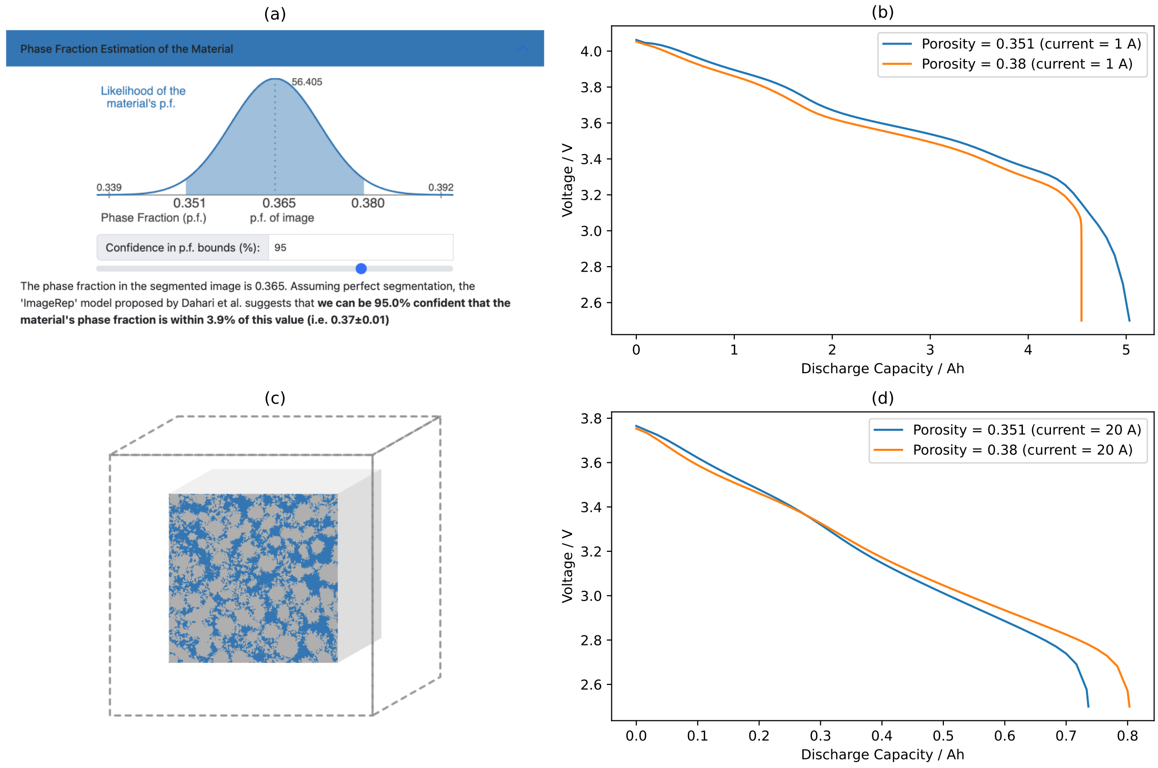

Figure 2: Impact of phase fraction uncertainty on battery performance. (a) presents the 95% confidence interval of the bulk material phase fraction of the 2nd calendered microstructure from [7]. (b) and (d) presents the discharge curves of two NMC cathodes using Pybamm [8] with the default NMC cathode parameters that originate from [9] by only changing the porosity values, the two bounding phase fraction values of the confidence interval (i.e. 0.351 and 0.380). (b) presents the discharge curves at low current ($\sim C/5$) and (d) at high current ($\sim 4C$). (c) Presents the volume that its phase fraction is predicted to be within only 2% of the material phase fraction with 95% confidence, instead of 3.9% with the current volume, a 392^3 voxels volume instead of 252^3 voxels volume, at the same resolution.

5.5 Validation including only std prediction step

Table 2 shows all of the validation results. The label 'only std' is used to indicate that the prediction was made solely by estimating the standard deviation of the sample using the Two-Point Correlation function, without the data-driven correction step presented in the end of Section 3. The results presented here also show why this step helps prediction accuracy.

6 Discussion SI

6.1 Impact of phase fraction uncertainty on battery performance

Figure 2 shows the impact of phase fraction uncertainty on battery performance, and how the model predict the image size needed for a specific smaller uncertainty.

6.2 Assessing image unrepresentativeness

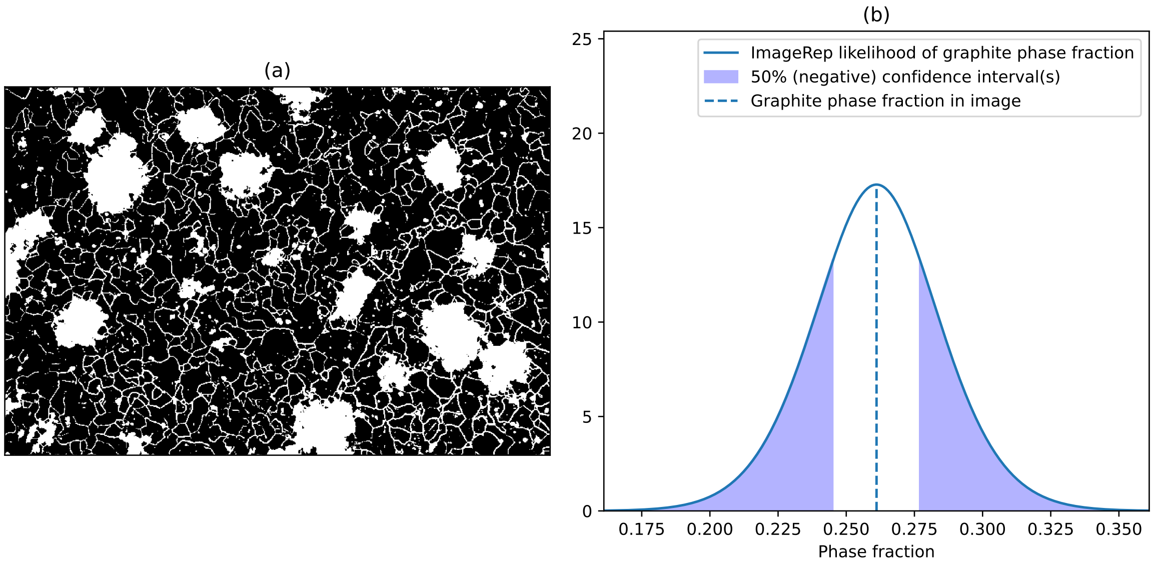

Figure 3: (a) Micrograph 368 from DoITPoMS of Blackheart cast iron [10] (after microlib [11] segmentation), showing the graphite phase along with ferrite grain boundaries. (b) Presents the 50% tails of the likelihood distribution of the true phase fraction of the material. There is a 50% probability (a coin toss) that the actual material phase fraction deviates by more than 6% from the apparent phase fraction in the image, indicating that the image may be far from being representative of the material phase fraction.

References

[1] Adams, B. L., Kalidindi, S. R. & Fullwood, D. T. *Microstructure sensitive design for performance optimization* (Butterworth-Heinemann, 2012).

[2] Hsu, T. *et al.* Mesoscale characterization of local property distributions in heterogeneous electrodes. *Journal of Power Sources* **386**, 1–9 (2018).

[3] Squires, I., Dahari, A., Cooper, S. J. & Kench, S. Artefact removal from micrographs with deep learning based inpainting. *Digital Discovery* **2**, 316–326 (2023).

[4] Gostick, J. T. *et al.* Porespy: A python toolkit for quantitative analysis of porous media images. *Journal of Open Source Software* **4**, 1296 (2019).

[5] Kanit, T., Forest, S., Galliet, I., Mounoury, V. & Jeulin, D. Determination of the size of the representative volume element for random composites: statistical and numerical approach. *International Journal of solids and structures* **40**, 3647–3679 (2003).

[6] Kanit, T. *et al.* Apparent and effective physical properties of heterogeneous materials: Representativity of samples of two materials from food industry. *Computer Methods in Applied Mechanics and Engineering* **195**, 3960–3982 (2006).

[7] Usseglio-Viretta, F. L. *et al.* Resolving the discrepancy in tortuosity factor estimation for li-ion battery electrodes through micro-macro modeling and experiment. *Journal of The Electrochemical Society* **165**, A3403–A3426 (2018).

[8] Sulzer, V., Marquis, S. G., Timms, R., Robinson, M. & Chapman, S. J. Python battery mathematical modelling (pybamm). *Journal of Open Research Software* **9** (2021).

[9] Chen, C.-H. *et al.* Development of experimental techniques for parameterization of multi-scale lithium-ion battery models. *Journal of The Electrochemical Society* **167**, 080534 (2020).

[10] Barber, Z., Leake, J. & Clyne, T. The doitpoms project-a web-based initiative for teaching and learning materials science. *Journal of Materials Education* **29**, 7 (2007).

[11] Kench, S., Squires, I., Dahari, A. & Cooper, S. J. Microlib: A library of 3d microstructures generated from 2d micrographs using slicegan. *Scientific Data* **9**, 645 (2022).
